# Wideband Waveform Design for Distributed Precision Jamming

**DOI:** 10.3390/e25030496

**Published:** 2023-03-13

**Authors:** Kedi Zhang, Qingsong Zhou, Jing Wang, Chao Huang, Zhongping Yang, Jianyun Zhang

**Affiliations:** 1 College of Electronic Engineering, National University of Defense Technology, Hefei 230000, China; zhangkedi10@nudt.edu.cn (K.Z.); 18226658179@163.com (C.H.); yangzhongping14@nudt.edu.cn (Z.Y.); zhangjianyun17@nudt.edu.cn (J.Z.); 2 College of Computer, National University of Defense Technology, Changsha 410073, China; wangjing@nudt.edu.cn

**Keywords:** distributed precision jamming, multiple-input and multiple-output system, wideband jamming waveform design, majorization minimization algorithm

## Abstract

Precision electronic warfare is a hot direction for future jamming technology development, and distributed precision jamming (DIPJ) is one of its typical application scenarios. The task objective of DIPJ is to design jamming waveforms so that the jamming energy generated by a set of ultra-sparse array transmitters can be focused in the jamming region of interest while being suppressed in other specific protected regions, which can be viewed as a distributed multiple-input and multiple-output system waveform design problem under a three-dimensional scenario. This paper extends the jamming signal model in DIPJ from narrowband to wideband based on previous work to address a broader range of jamming tasks. After extending the model to wideband signals, a method based on the traditional maximum total energy difference optimization objective is first given for comparison. A wideband jamming waveform design method based on the majorization minimization algorithm with the desired power spectrum matching as the optimization target is designed for the problem that the maximum energy difference method cannot focus energy well in the jamming region. The simulation results show that the presented method can make the jamming energy well concentrated in the target region and evenly distributed over the whole bandwidth, while the energy in the whole bandwidth is suppressed in the protected region.

## 1. Introduction

The development of military science and technology, especially electronic warfare technology, is closely related to the development of information technology [1]. At the same time, electronic warfare means are the only way to offset the technical superiority of the opponent in terms of information and technology [2], making it an area of widespread concern in the direction of information technology [3]. In order to overcome the characteristics of traditional oppressive jamming, such as low efficiency, ease to counteract, and the ease with which friendly targets can be accidentally injured [4], the Strategic Technology Office (STO) of the U.S. Defense Advanced Research Projects Agency (DARPA) proposed precision electronic warfare (PREW) in 2009 [5]. PREW aims to surgically perform precision jamming in scenarios as small as city streets by using a self-organizing, ultra-sparse array of low-cost, low-power, small and lightweight distributed airborne and ground-based nodes [6].

### 1.1. Related Work

With the increasing advancement of small Unmanned Aerial Vehicle (UAV) technology in terms of positioning [7], communication [8], and networking [9], it is possible to use multiple UAVs to cooperate in jamming tasks. In a typical application of PREW, multiple UAVs carry jamming transmitters to complete distributed precision jamming (DIPJ), the key technology of which is focused energy delivery (FED) [10]. Previous energy focusing methods based on beam-forming, such as the delay-and-sum (DAS)-based phased array [11,12], can only produce a cone-shaped high-energy field directed at a predetermined angle, leading to the jamming energy not necessarily accurately covering the preset jamming area, and the interference energy easily leaking into non-target areas, thus increasing the risk of electromagnetic damage to friendly devices [13].

Inspired by the need to focus emitter energy to kill tumor cells while not harming healthy tissues in breast cancer treatments [14], DIPJ precisely focuses energy within a given jamming region by forming an ultra-sparse array of multiple transmitters in space. This does not mean that DIPJ uses an energy level in the focus area that is higher than traditional techniques such as DAS 10, but it requires accurate coverage of the given target area. At the same time, because the transmitter usually adopts an ultra-sparse array arrangement, other high-energy regions will be generated in regions outside the jamming region due to the grating lobe effect [15]. This requires that DIPJ also minimize jamming energy at locations in which friendly or other detection devices may be present.

Subsequent research in the field of DIPJ has focused on how to suppress the grating lobe effect in other areas of the space besides the areas that require protection [16]. By adjusting the regularization factor from the l-l norm to the infinite norm, the highest grating lobe points in the space were further inhibited [13,15]. In order to increase the degree of freedom of the design signal, improved models for optimizing the transmitter position [17] and adding multiple transmitter array elements to each transmitter [18] have also been proposed. However, there are two major problems in the literature described above. One is that the signal designed by the method only considers a single moment. If the same signal is forwarded repeatedly, even if the energy focus can be realized in space, the working bandwidth of the interfered target may not be covered well in some cases. Secondly, the optimization criteria are based on the maximum total energy difference (MTED) between the jamming area and the protected area, which is closer to the traditional jamming method of roughly placed energy, resulting in some pre-defined jamming areas not possessing a good energy focus effect. 

In order to overcome this problem, a jamming waveform method for DIPJ application scenarios was proposed [19]. In this method, the covariance matrix of the wide-sense stationary transmitted jamming signal is modeled as a semi-finite programming (SDP) problem [10], and the sequential cone programming (SOCP) algorithm [20] is used to design the jamming waveform using the time average instead of the statistical average in order to match the covariance matrix. At the same time, the autocorrelation function of the synthetic waveform emitted by each transmitter in the jamming area is constrained to be as close as possible to the delta function, so that the jamming energy is able to cover the working bandwidth of the target as much as possible. Another understanding that has been acquired is that the best oppressive jamming waveform is that with the greatest degree of uncertainty [21], and the highest degree of randomness with the jamming waveform autocorrelation function is demonstrated by the delta function.

However, all these DIPJ models address narrowband signals that assume that the baseband signal is delay-free in the far field because the baseband bandwidth is negligible relative to the carrier frequency. When the jamming target uses a wideband signal or the target operating bandwidth is uncertain, a wideband signal needs to be used to jam the possible operating bandwidth of the target, and the jamming effect must be reexamined on the basis of the wideband model. 

Due to the sensitivity of its military application background, jamming waveform design has rarely been described, and is usually carried out aiming at a specific application system [22,23]. Another relatively new hot area is jamming waveform design in cognitive electronic warfare [24]. However, the background of this other form of jamming waveform design is different from in the case of DIPJ applications, leading to different optimization objectives.

The DIPJ application itself can be seen as a MIMO (Multiple-Input Multiple-Output) system [25,26] in a three-dimensional scenario with a distributed transmitter arrangement. The waveform design problem of the transmitted beampattern matching design in MIMO radar [27] is also concerned with the energy focusing problem of MIMO systems; in particular, its mathematical model on wideband signals [28,29] can serve as a useful reference for the DISP wideband jamming waveform design problem, but there are still some differences between them. For example, MIMO radar expects the energy focus to be in the spatial direction, and therefore does not take attenuation into account in its guidance vector, which is more like traditional oppressive jamming with rough energy projection in certain directions. MIMO radar transmitters usually use array structures with regular time delays, because the radar system is focusing on detection [30]. However, in the jamming task, it is not necessary to consider the problem of the receipt of echoes of the transmitted waveform. Combined with distributed application, the transmitter adopts an ultra-sparse array in DIPJ.

### 1.2. Major Contributions

In this paper, we propose a method for solving the jamming waveform design problem for wideband DISP. The main contributions are as follows:

- Previous research on DIPJ based on a mathematical model of the narrowband signal is extended to a wideband signal to adapt it to a wider range of applications.
- A majorization minimization (MM) algorithm [31] is given for wideband jamming signal design based on the MTED objective. This type of optimization objective, based on the MTED between the jamming region and the protected region, has been widely used in previous studies of DIPJ [13,16,17,18]. However, it has the disadvantage of not being able to precisely control the jamming energy to remain high throughout in the target operating bandwidth, and we mainly use it for comparison with the desired power spectrum matching (DPSM) method.
- In order to better achieve precision jamming, we propose a jamming waveform design method based on DPSM. It firstly determines an approximate expected value of the power density to be matched on the basis of a simple semi-definite programming (SDP) problem. Then, a complete derivation and algorithmic complexity analysis are given for solving a nonconvex problem containing quartic terms using the MM algorithm, including a fast approximation for solving the maximum eigenvalue of the matrix.
- The results of the above algorithm are analyzed by simulating a typical DIPJ scenario. The quantitative results show that, compared with the method based on MTED, the DPSM method is able to design a wideband jamming waveform that is able to more accurately form a high-power focus in the jamming region over the entire bandwidth while suppressing power over the entire bandwidth of the protected region. However, compared with the previously inapplicable narrowband-based method, the MTED method can still be used for the wideband jamming waveform design of DIPJ, and it offers lower computational complexity than DPSM.

## 2. Wideband DIPJ Problem Formulation

**Figure 1:**
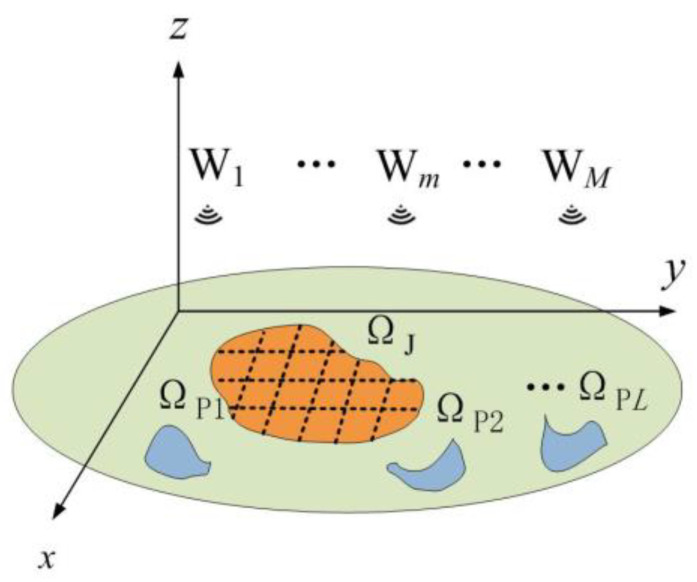
Typical DIPJ application scenario.

A schematic diagram of the DIPJ application scenario is presented in Figure 1. $W_{m}\left(m=1,\;2,\;\ldots ,M\right)$ represents the total of *M* transmitters carried by the UAVs. It is necessary for the electromagnetic power of the transmitter to be focused in the jamming area Ω_J_ and limited to *L* in the protected areas $\Omega_{P}=\Omega_{P1}\cup \Omega_{P2}\cup \ldots \Omega_{PL}$. To facilitate the computational simulation, the scene is discretized into grid cells, and the geometric centroids of the grid cells are used to represent the locations of regions. The jamming area Ω_J_ and the protected area Ω_P_ consist of the grids *I* and *J*, which can be constructed using sets represented as $U=\{\sigma_{i}\in \Omega_{J},i=1,2,\ldots ,I\}$ and $V=\{\sigma_{j}\in \Omega_{P},j=1,2,\ldots ,J\}$, respectively. 

Similar to previous research, we assume that all positioning information is accurate and there is no positioning error, and that the transmitter timing is unified and there are no problems with signal delay between transmitters. 

The far-field signal at any point σ in the region is

(1) $$s_{\sigma }(t)=\sum_{m=1}^{M}x_{m}(t-\tau_{m}(\sigma ))\frac{e^{j2\pi f_{0}(t-\tau_{m}(\sigma ))}}{\left|l_{m}-\sigma \right|}.$$

Here, ***l****_m_* is the location of the *m*-th transmitter, so that $\left|l_{m}-\sigma \right|$ is the transmission distance; dividing by this indicates the attenuation of amplitude. *x_m_* is the baseband signal, $\tau_{m}(\sigma )=\left|l_{m}-\sigma \right|/c$ is the time delay, and *f*_0_ is the carrier frequency. Because the baseband delay is small compared to the carrier in the narrowband model, and can therefore be directly ignored, the synthetic power at the grid cell σ can be expressed as follows [10]:

(2) $$P_{\sigma }=a{\left(\sigma \right)}^{H}Ra(\sigma ),a(\sigma )={\left[\frac{e^{j2\pi f_{0}\tau_{1}(\sigma )}}{\left|l_{1}-\sigma \right|},\ldots ,\frac{e^{j2\pi f_{0}\tau_{M}(\sigma )}}{\left|l_{M}-\sigma \right|}\right]}^{T}.$$

Here, ***R*** is the covariance matrix of the baseband signal. The symbols (.)^T^ and (.)^H^ denote the transposition and the conjugate transposition, respectively. However, when the delay of the baseband signal is not negligible (i.e., when the baseband bandwidth of the wideband signal is not less than 10% of the carrier frequency), Equation (2) does not hold, and analysis needs to be performed from the frequency domain.

The inverse Fourier transform of the baseband signal by the *m*-th transmitter can be written as

(3) $$x_{m}(t)=\int_{-B/2}^{B/2}y_{m}(f)e^{j2\pi ft}df,$$

where *B* is the baseband bandwidth and $y_{m}(f)$ is the Fourier transform of the baseband signal by the *m*-th transmitter. Combined with Equation (1), this yields [28]

(4) $$s_{\sigma }(t)=\int_{-B/2}^{B/2}\sum_{m=1}^{M}\frac{1}{\left|l_{m}-\sigma \right|}y_{m}(f)e^{-j2\pi (f_{0}+f)\tau_{m}(\sigma )}e^{j2\pi (f+f_{0})t}df.$$

The power spectrum at grid cell σ and frequency *f* is

(5) $$P_{\sigma }(f)={\left|a{\left(\sigma ,f\right)}^{H}y(f)\right|}^{2},$$

where

(6) $$y(f)={\left[y_{1}(f),y_{2}(f)\;\ldots y_{m}(f)\right]}^{T}$$

and

(7) $$a(\sigma ,f)=={\left[\frac{e^{j2\pi (f+f_{0})\tau_{1}(\sigma )}}{{\|l}_{1}-\sigma \|},\frac{e^{j2\pi (f+f_{0})\tau_{2}(\sigma )}}{{\|l}_{2}-\sigma \|},\ldots ,\frac{e^{j2\pi (f+f_{0})\tau_{m}(\sigma )}}{{\|l}_{m}-\sigma \|}\right]}^{T}.$$

If the baseband is a digitally sampled discrete signal with a total sequence length of *N*, then using the discrete Fourier transform (DFT) [32], the following can be obtained:

(8) $$y(p)=Xk_{p},\;p=-N/2,\ldots 1,\ldots N/2-1$$

and

(9) X=[x(1) …x(n)…x(N)].

In Equation (9), ***x***(*n*) is an *M* × 1-dimensional vector representing the sampling of *M* transmitters at moment *n*. Additionally,

(10) $$k_{p}={\left[1,e^{-j2\pi (p-1)/N},\ldots ,e^{-j2\pi (N-1)(p-1)/N}\right]}^{T}/\sqrt{N},\;p=-N/2,\ldots 1,\ldots N/2-1,$$

is used with to denote the DFT. Here, it is considered that the number of samples in the frequency domain is also *N*, which is the same as in the time domain. The discrete form of Equation (7) can be written as

(11) $$a(\sigma ,f_{p}),f_{p}=p/(NT_{s}),$$

where *T_s_ =* 1/*B* is the sampled period of the baseband. The power spectrum at grid cell σ with frequency point *f_p_* can be expressed as

(12) $$P_{{}_{\sigma },f_{p}}={\left|a{\left(\sigma ,f_{p}\right)}^{H}Xk_{p}\right|}^{2}.$$

Unlike the narrowband signal model, which requires consideration of the frequency domain characteristics after optimizing the space domain energy distribution [26], the power spectral density obtained in Equation (12) is the result of a joint optimization in the space and frequency domains. This is very much in line with the need for the jamming task to cover the target operating bandwidth, and in the next section, we will describe the method for designing the jamming waveform.

## 3. Jamming Waveform Design Optimization Algorithm

In this section, we will give the method for solving the jamming waveform using the MM algorithm for two optimization objectives. The first uses the MTED between the jamming region and the protected region as the optimization objective. This optimization criterion has been widely used in previous narrowband models, but it does not distribute the energy in each frequency band well when employing wideband models. The second uses DPSM as the optimization objective, which is more suitable for wideband signal models.

### 3.1. Jamming Waveform Design with MTED

The DIPJ problem with MTED as the optimization objective can be written in the following form [16]:

(13) $$\underset{X}{\max}\sum_{\sigma \in U}^{}\sum_{p=-N/2}^{N-1}{\left|a{\left(\sigma ,f_{p}\right)}^{H}Xk_{p}\right|}^{2}-\sum_{\sigma \in V}^{}\sum_{p=-N/2}^{N-1}{\left|a{\left(\sigma ,f_{p}\right)}^{H}Xk_{p}\right|}^{2}.$$

In order to improve the energy utilization efficiency of the transmitter power amplifier, a constant-modulus constraint is applied to the transmitted signal. Without a loss of generality, we set the waveform modulus constant to 1; then, we can use the MM algorithm to solve it. First, Equation (13) can be rewritten as

(14) $$\begin{array}{l} \underset{x_{v}}{\max}\sum_{\sigma \in U}^{}\sum_{p=-N/2}^{N-1}{\left|x_{v}{}^{H}A(\sigma ,f_{p})k^{*}{}_{p}\right|}^{2}-\sum_{\sigma \in V}^{}\sum_{p=-N/2}^{N-1}{\left|x_{v}{}^{H}A(\sigma ,f_{p})k^{*}{}_{p}\right|}^{2}\\ s.t.\left|x_{v}(t)\right|=1,\;t=1,\ldots ,MN, \end{array}$$

where

(15) $$A(\sigma ,f_{p})=I_{N}⊗a(\sigma ,f_{p})\;\;x_{v}=\mathrm{vec}(X)$$

and ⊗ is the Kronecker product, (.)* denotes the conjugate, ***I****_N_* is the *N*-dimensional unit matrix, and vec(.) denotes the vectorization of the matrix by columns. Expanding Equation (14), we have

(16) $$\begin{array}{l} \underset{x_{v}}{\max}\;x_{v}{}^{H}\left(\sum_{\sigma \in U}^{}\sum_{p=-N/2}^{N-1}F(\sigma ,f_{p})-\sum_{\sigma \in V}^{}\sum_{p=-N/2}^{N-1}F(\sigma ,f_{p})\right)x_{v}\\ s.t.\left|x_{v}(t)\right|=1,\;t=1,\ldots ,MN, \end{array}$$

where

(17) $$F(\sigma ,f_{p})=A(\sigma ,f_{p})k_{p}{}^{*}k_{p}{}^{T}A{\left(\sigma ,f_{p}\right)}^{H}.$$

Considering $\sum_{\sigma \in U}^{}\sum_{p=-N/2}^{N-1}F(\sigma ,f_{p})-\sum_{\sigma \in V}^{}\sum_{p=-N/2}^{N-1}F(\sigma ,f_{p})$ as a matrix ***R***, the Equation (16) is equivalent to the following equation:

(18) $$\begin{array}{l} \underset{x_{v}}{\min}\;x_{v}{}^{H}\left(\beta I_{MN}-R\right)x_{v}\\ s.t.\;\left|x_{v}(t)\right|=1,\;t=1,\ldots ,MN, \end{array}$$

where β is a given constant, and to ensure $\left(\beta I_{MN}-R\right)=R\prime \underline{≻}0$, β should take a value greater than the maximum eigenvalue of R.

The MM algorithm refers to a class of iterative methods dealing with hard optimization problems. Equation (18) is a quadratic minimization problem, which can be solved iteratively using the MM algorithm. Based on the majorant of the quadratic form derived in previous studies [31], the MM algorithm solves Equation (18) for the *z* + 1-th iteration by solving the following problem:

(19) minxv xvHλIMNxv+2Re(xvH(R′−λIMN)xv(z))+(xv(z))H(λIMN−R′)xv(z)s.t. |xv(t)|=1, t=1,…,MN,

where $x_{v}{}^{\left(z\right)}$ is the result of the *z-*th iteration and λ is the maximum eigenvalue of R′. Because the modulus of the signal is constant, the quadratic terms in Equation (19) with respect to both $x_{v}$ and $x_{v}{}^{\left(z\right)}$ are constants. Then, Equation (19) can be further rewritten as

(20) minxv Re(xvH(R′−λIMN)xv(z))s.t. |xv(t)|=1, t=1,…,MN,

where the Re(.) denotes the real part. Equation (20) is thus essentially equivalent to

(21) $$\begin{array}{l} \underset{x_{v}}{\min}\;{\left\|x_{v}+\left(R\prime -\lambda I_{MN}\right)x_{v}{}^{\left(z\right)}\right\|}_{2}\\ s.t.\;\left|x_{v}(t)\right|=1,\;t=1,\ldots ,MN. \end{array}$$

The closed solution of Equation (21) is

(22) $$x_{v}{}^{\left(z+1\right)}=e^{j\arg\left((-R\prime -\lambda I_{MN})x_{v}{}^{\left(z\right)}\right)},$$

where arg(.) denotes the extraction phase operation. Because the convergence [32] of the MM algorithm is demonstrated, we can set a random constant-modulus waveform as the initial value and solving Equation (22) iteratively until the convergence threshold ε is reached, which yields the jamming waveform. However, following our tests (which will be shown in the next part of the simulation), this algorithm can be used to design a jamming waveform that is capable of creating the spatial energy effect needed by DIPJ, while concentrating the energy in the frequency domain at one frequency point, since the algorithm only cares about the difference in total energy at all frequency points. This is a new problem encountered in the wideband model that does not exist in the narrowband model. 

In order to improve the effect of the design jamming waveform, we first optimize *N* jamming waveforms by targeting the MTED at a single frequency point *p*. 

$x_{p}$ is used to denote the jamming waveform obtained by optimizing the MTED target at the frequency *p*; then, the optimization objective in Equation (14) is replaced as follows:

(23) $$\begin{array}{l} \underset{x_{p}}{\min}\;x_{p}{}^{H}\left(\beta \prime I_{MN}-R\prime \prime \right)x_{p}\\ s.t.\;\left|x_{p}(t)\right|=1,\;t=1,\ldots ,MN, \end{array}$$

where $R\prime \prime =\sum_{\sigma \in U}^{}F(\sigma ,p)-\sum_{\sigma \in V}^{}F(\sigma ,p)$ and β′ is a value greater than the maximum eigenvalue of R″. The MM algorithm is the same as that used to solve the problem. The *N* obtained waveforms are processed as follows to synthesize a final jamming waveform.
**Algorithm 1:** MTED method**Input:**$\;a(\sigma ,f_{p}),k_{p},\varepsilon ,M,N$**For** *p* = −*N*/2 to *N*/2 − 1**Initialize:**$\;x_{p}{}^{\left(0\right)},z=0$1: Calculate R″ according to $a(\sigma ,f_{p}),k_{p}$2: Calculate β′ according to R″.3: Calculate λ according to $\left(\beta \prime I_{MN}-R\prime \prime \right)$.**While** converges threshold ε is not reached **do**4: Calculate $x_{p}{}^{\left(z+1\right)}$ according to Equation (22).5: *z* = *z*+1.**End while****End for**6: Calculate $x_{v}$ according to Equation (24)

(24) $$x_{v}=e^{j\;\arg(\sum_{p=-N/2}^{N/2-1}x_{p})}$$

The overall algorithm flow is shown in Algorithm 1. For the sake of description, we will call this the MTED method. According to the computational complexity of matrix multiplication, computing R″ requires $O(2(I+J)MN^{2})$, and calculating the maximum eigenvalues of β′ and λ requires O(2MN). The computational complexity of each iterative $O(M^{2}N^{2})$ is linear with the number of iterations $\tilde{T}$. There are *N* waveforms that need to be computed, so the total algorithm complexity of the MTED method is $O(2(I+J)MN^{3}+2MN^{2}+\tilde{T}M^{2}N^{3})$.

The waveforms obtained by the MTED method greatly improves the energy distribution in the frequency domain, as will be shown in detail in the simulation part. However, in order to further improve the energy distribution in the frequency domain of the jamming waveform, we propose an optimization target method based on DPSM. 

### 3.2. Jamming Waveform Design with DPSM

#### 3.2.1. Estimated Desired Power Spectrum

The idea of DPSM is to let each point on the power spectrum match a desired value so that it can be more accurately controlled in the frequency domain. In DIPJ applications, the operating band of the target device is equal to the bandwidth of the wideband jamming waveform or lies in an unknown segment of the jamming waveform bandwidth. On the basis of this assumption, we want to design the jamming waveform with the highest possible total energy of the power spectrum at each grid cell of the jamming region, and the shape of the power spectrum resembles a gate function uniformly distributed over the entire bandwidth. In two-dimensional MIMO systems such as MIMO radar waveforms, similar effects are usually determined by beam pattern matching [27], because there are only two types of regions: the main lobe region, which needs to focus energy, and the side lobe region, which inhibits energy. However, because there are other regions (the green area in Figure 1) besides the jamming and protected regions, we cannot match them according to the desired beam pattern, as is possible in the MIMO radar waveform design. Note that Equation (12) can be written as

(25) $$\begin{array}{ll} P_{\sigma ,f_{p}} & =a{\left(\sigma ,f_{p}\right)}^{H}y(p)y{\left(p\right)}^{H}a(\sigma ,f_{p})\\ & =a{\left(\sigma ,f_{p}\right)}^{H}S_{p}a(\sigma ,f_{p}), \end{array}$$

where ***S****_p_* is the cross-spectral density matrix [33] of the baseband and ***y***(*p*) is the frequency domain signal transformed by Fourier transform. A reasonable desired power spectrum target can be obtained by optimizing ***S****_p_*. The following optimization problem is given:

(26) $$\begin{array}{l} \max_{S_{p}}\;\min_{\mu }\;\mu \\ s.t.\;a{\left(\sigma ,f_{p}\right)}^{H}S_{p}a(\sigma ,f_{p})\geq \mu \\ \;\;S_{p}\underline{≻}0\\ \;\;diag{\left(S_{p}\right)}_{mm}=1m=1,\ldots ,M,\;\sigma \in U. \end{array}$$

$a{\left(\sigma ,f_{p}\right)}^{H}S_{p}a(\sigma ,f_{p})\geq \mu$ indicates that the minimum power at points in the jamming region at frequency *p* is greater than *μ* (*μ* is also the quantity to be optimized). $\;S_{p}\underline{≻}0$ constrains this to be a semi-positive definite matrix. $diag{\left(S_{p}\right)}_{mm}=1\;$ indicates that the diagonal elements are all 1, which is equivalent to dividing the total *NM* energy of the constant-mode signal evenly into *N* parts, and the total energy at each frequency point is *M*. This simultaneously ensures that the total energy emitted by each UAV is equal. Equation (26) is a convex problem that can be solved directly using the CVX toolbox [34]. Notice that ***y***(*p*)***y***(*p*)^H^ has rank one, and the ***S****_p_* optimized by Equation (26) is full rank, so the idea of continuing optimization after ***S****_p_* to obtain ***y***(*p*) to obtain the jamming waveform is not achievable [28].

Then, ***S****_p_* is used to find the grid cell $\sigma_{i}$ with the greatest power spectrum value in the jamming region at each frequency point *p*.

(27) $$EJ(p)=\max\left(a{\left(\sigma_{i},f_{p}\right)}^{H}S_{p}a(\sigma_{i},f_{p}),\;\sigma_{i}\in U\right)$$

The desired power spectrum obtained by Equation (27) is a highly suitable matching target. This is because, in Equation (26), the power spectrum of each grid cell in the entire jamming region is optimized to be as large as possible after fixing a certain frequency point *p*. When we then design the jamming waveform using the same optimization target for the entire frequency band, the power spectrum of the jamming waveform can be close to the desired power spectrum, but cannot exceed it, as will be verified in the simulation section.

#### 3.2.2. Mathematical Derivation of Algorithm

The new optimization objective is to make the power spectrum of the designed jamming waveform as close as possible to the desired power spectrum in the jamming region, and as low as possible in the protected region. Based on the principle of least squares, the new optimization function is

(28) $$\begin{array}{l} \underset{x_{v}}{\min}\;w_{1}\sum_{\sigma \in U}^{}\sum_{p=-N/2}^{N-1}{\left({\left|x_{v}{}^{H}A(\sigma ,f_{p})k^{*}{}_{p}\right|}^{2}-EJ(p)\right)}^{2}+w_{2}\sum_{\sigma \in V}^{}\sum_{p=-N/2}^{N-1}{\left|x_{v}{}^{H}A(\sigma ,f_{p})k^{*}{}_{p}\right|}^{2}\\ s.t.\;\left|x_{v}(t)\right|=1,\;t=1,\ldots ,MN. \end{array}$$

Equation (28) is a multi-objective optimization problem, where *w*_1_, *w*_2_ are scaling factors used to adjust the weights of the two optimization objectives. Equation (28) contains a non-convex quartic term, and we need to vary the form in order to iterate the solution using the MM algorithm. Expanding Equation (28) for the quartic term, we obtain the following:

(29) $$w_{1}\sum_{\sigma =U}^{}\sum_{p=-N/2}^{N/2-1}{\left|{\left(x_{v}\right)}^{H}F(\sigma ,f_{p})x_{v}\right|}^{4}{=\mathrm{vec}}^{H}(x_{v}x_{v}{}^{H})\left(w_{1}\sum_{\sigma =U}^{}\sum_{p=-N/2}^{N/2-1}\mathrm{vec}\left(F(\sigma ,f_{p})\right){\mathrm{vec}}^{H}\left(F(\sigma ,f_{p})\right)\right)\mathrm{vec}(x_{v}x_{v}{}^{H}).$$

Here, we apply the formula $\mathrm{tr}(A^{H}B{)=\mathrm{vec}}^{H}(A)\mathrm{vec}(B)$, where tr(.) takes the trace of the matrix.

Let $P=w_{1}\sum_{\sigma =U}^{}\sum_{p=-N/2}^{N/2-1}\mathrm{vec}\left(F(\sigma ,f_{p})\right){\mathrm{vec}}^{H}\left(F(\sigma ,f_{p})\right)$; then, the majorant of the quadratic form is applied and the constant term is omitted. The MM algorithm solves the quartic terms contained in Equation (29) in the *z* + 1th iteration by solving the following problem:

(30) minxv 2Re(vecH(xvxvH)(P−λ1IMN)vecH(xv(z)(xv(z))H))s.t. |xv(t)|=1, t=1,…,MN,

where $\lambda_{1}$ is the maximum eigenvalue of ***P***. The dimensionality of the ***P*** matrix is large, and solving its eigenvalues is slow (in our simulation scenario below, the dimensionality of the ***P*** matrix exceeds the upper limit of memory that can be stored); here, we give a fast approximate solving method. According to the trigonometric inequality, we have

(31) $$\lambda_{\max}\left(P\right)\leq \sum_{\sigma_{i}=1}^{I}\lambda_{\max}\left(P_{\sigma_{i}}\right),$$

where $\lambda_{\max}\left(.\right)$ denotes the maximum eigenvalue and $P_{\sigma_{i}}=w_{1}\sum_{p=-N/2}^{N/2-1}\mathrm{vec}\left(F(\sigma_{i},f_{p})\right){\mathrm{vec}}^{H}\left(F(\sigma_{i},f_{p})\right)$. According to the definition of eigenvalues, when *p* is a finite number, for any $k_{j}\in [-N/2,\ldots ,N/2-1]$, we have [35]

(32) $$\begin{array}{l} P_{\sigma_{i}}\mathrm{vec}\left(F(\sigma_{i},f_{kj})\right)=\sum_{p=-N/2}^{N/2-1}\mathrm{vec}\left(F(\sigma_{i},f_{p})\right){\mathrm{vec}}^{H}\left(F(\sigma_{i},f_{p})\right)\mathrm{vec}\left(F(\sigma_{i},f_{k_{j}})\right)\\ =\sum_{p=-N/2}^{N/2-1}tr\left(k_{p}{}^{*}k_{p}{}^{T}a{\left(\sigma_{i},f_{p}\right)}^{H}a(\sigma_{i},f_{p})k_{p}{}^{*}k_{p}{}^{T}a{\left(\sigma_{i},f_{p}\right)}^{H}a(\sigma_{i},f_{p})\right)\mathrm{vec}\left(F(\sigma_{i},f_{k_{j}})\right)\\ \leq N\max{\left(a{\left(\sigma_{i},f_{p}\right)}^{H}a(\sigma_{i},f_{p}),p\in [-N/2,\ldots ,N/2-1]\right)}^{2}\mathrm{vec}\left(F(\sigma ,f_{k_{j}})\right). \end{array}$$

The following inequation is obtained:

(33) $$\lambda_{1}=\lambda_{\max}\left(P\right)\leq \sum_{\sigma_{i}=1}^{I}\lambda_{\max}\left(P_{\sigma_{i}}\right)\leq \sum_{\sigma_{i}=1}^{I}N\max{\left(a{\left(\sigma_{i},f_{p}\right)}^{H}a(\sigma_{i},f_{p}),p\in [-N/2,\ldots ,N/2-1]\right)}^{2}.$$

Since applying the majorant of the quadratic form requires a semi-positive definite matrix larger than ***P***, using a number larger than the maximum eigenvalue naturally satisfies the requirement. This allows $\lambda_{1}$ to be obtained by quick calculation with the modal value of $a(\sigma ,f_{p})$.

By expanding Equation (30), we obtain

(34) 2Re(vecH(xvxvH)(P−λ1IMN)vec(xv(z)(xv(z))H))=2Re(w1∑σ=U∑p=−N/2N/2−1tr(xvHF(σ,fp)xv)tr((xv(z))HF(σ,fp)Hxv(z)))−2λ1xvHxv(z)(xv(z))Hxv=xvH(2w1∑σ=U∑p=−N/2N/2−1((xv(z))HF(σ,fp)Hxv(z)F(σ,fp))−2λ1xv(z)(xv(z))H)xv.

**Algorithm 2:** DPSM method**Input:**$\;w_{1},w_{2},a(\sigma ,f_{p}),k_{p},\varepsilon ,M,N$.
**Initialize:**

$\;x_{v}{}^{\left(0\right)},z=0$

1: Calculate EJ(p) according to Equations (26) and (27).2: Calculate $\lambda_{1}$ according to Equation (33).3: **While** converges threshold ε is not reached **do**4: Calculate $Q_{1}\;\mathrm{and}\;Q_{2}$ according to Equation (37).6: Calculate $\lambda_{2}\;\mathrm{and}\;\lambda_{3}$ according to Equation $Q_{1}\;\mathrm{and}\;Q_{2}$.7: Calculate $x_{v}{}^{\left(z+1\right)}$ according to Equation (36).8: *z* = *z*+1.9: **End while**

After omitting the constant term, applying the MM algorithm to solve the *z* + 1-th iteration of Equation (28) is equivalent to solving the following problem:

(35) $$\begin{array}{l} \underset{x_{v}}{\min}\;x_{v}{}^{H}\left(2w_{1}\sum_{\sigma =U}^{}\sum_{p=-N/2}^{N/2-1}\left(\left({\left({x_{v}}^{\left(z\right)}\right)}^{H}F{\left(\sigma ,f_{p}\right)}^{H}x_{v}{}^{\left(z\right)}-EJ(p)\right)F(\sigma ,f_{p})\right)-2\lambda_{1}x_{v}{}^{\left(z\right)}{\left(x_{v}{}^{\left(z\right)}\right)}^{H}\right)x_{v}\\ \;\;\;+w_{2}\sum_{\sigma \in V}^{}\sum_{p=-N/2}^{N/2-1}{\left|x_{v}{}^{H}A(\sigma ,f_{p})k^{*}{}_{p}\right|}^{2}\\ s.t.\;\left|x_{v}(t)\right|=1,\;t=1,\ldots ,MN. \end{array}$$

Note that the above equation is still quadratic with respect to $x_{v}$ and a majorant can be used again. As in the corollary presented in Section 3.1, the closed-form solution of Equation (35) is

(36) $$x_{v}{}^{\left(z+1\right)}=e^{j\arg\left(-2(Q_{1}-\lambda_{2}I_{MN}+Q_{2}-\lambda_{3}I_{MN})x_{v}{}^{\left(z\right)}\right)},$$

where *λ*_2_ and *λ*_3_ are the maximum eigenvalue of ***Q***_1_ and ***Q***_2_, respectively. ***Q***_1_ and ***Q***_2_ are

(37) $$\begin{array}{l} Q_{1}=w_{1}\sum_{\sigma =U}^{}\sum_{p=-N/2}^{N/2-1}\left(\left({\left(x_{v}{}^{\left(z\right)}\right)}^{H}F{\left(\sigma ,f_{p}\right)}^{H}x_{v}{}^{\left(z\right)}-EJ(p)\right)F(\sigma ,f_{p})\right)-\lambda_{1}x_{v}{}^{\left(z\right)}{\left(x_{v}{}^{\left(z\right)}\right)}^{H}\\ Q_{2}=w_{2}\sum_{\sigma =V}^{}\sum_{p=-N/2}^{N/2-1}F(\sigma ,f_{p}). \end{array}$$

**Algorithm 3:** MM Acceleration Strategy**Initialize:**${x_{v}}^{\left(0\right)},s^{\left(0\right)}={x_{v}}^{\left(0\right)},k=1,z=0$.**While** MM algorithm does not achieve converges threshold **do**1:$\;\mathrm{Take}\;s^{\left(k-1\right)}$ as the initial value and use DPSM algorithm for one iteration, and take the calculated result as $s^{\left(k\right)}$.2: *k* = *k*+ 1.**if** k < 3 go bank to step1
**else**
3: $r=s^{\left(1\right)}-s^{\left(0\right)}$.4: $g=s^{\left(2\right)}-s^{\left(1\right)}-r$.5: α=−‖r‖/‖g‖.6: $ss=e^{j\arg(s^{\left(0\right)}-2\alpha r-\alpha^{2}g)}$**While** use ss to calculate the value of the objective function in Equation (28), if it is greater than the value of the function calculated by $s^{\left(0\right)}$
**do**7: α=(α−1)/2.8: $ss=\exp^{j\;\arg(s^{\left(0\right)}-2\alpha r-\alpha^{2}g)}$
**End while**
9: *k* = 0, *z* = *z*+ 1.10:$s^{\left(0\right)}=ss$.11:${x_{v}}^{\left(z\right)}=ss$.
**End while**


The overall algorithm flow is shown in the Algorithm 2. For the sake of description, we will call this the DPSM method. Obtaining EJ(p) requires solving *N* SDP problems, and the computational complexity is $O(N{\left(M+\sqrt{I}\right)}^{3})$. The complexity of computing $\lambda_{1}$ is $O(IM^{2}N)$. The complexity of computing $Q_{1}\;\mathrm{and}\;Q_{2}$ is $O(2I(M^{2}N^{3}+MN^{3})+2JMN^{3}+M^{2}N^{2})$. The complexity of computing $\lambda_{2}\;,\lambda_{3}\;\mathrm{and}\;{x_{v}}^{\left(z+1\right)}$ is $O(2MN+M^{2}N^{2})$. 

By combining the number of iterations $\tilde{T}$, the total computational complexity of the DPSM method corresponds to $O(N{\left(M+\sqrt{I}\right)}^{3}+IM^{2}N+2\tilde{T}(I(M^{2}N^{3}+MN^{3})+JMN^{3}+M^{2}N^{2}+MN))$.

#### 3.2.3. Acceleration Strategy

The use of the majorant twice in Equations (30) and (36) may lead to a slow decline in the objective function with iteration of the MM algorithm. Since a large part of the computational complexity of the DPSM method is related to the number of iterations, we use squared iterative methods [36] to speed up the process. Here, only the operation flow is given in Algorithm 3, and interested readers should read the original reference. In the later part of the simulation, results will be given verifying that the squared iterative methods greatly speed up the convergence of the iterations. 

## 4. Simulation Verification and Results Discussion

### 4.1. Experimental Scenarios and Parameter Settings

**Figure 2:**
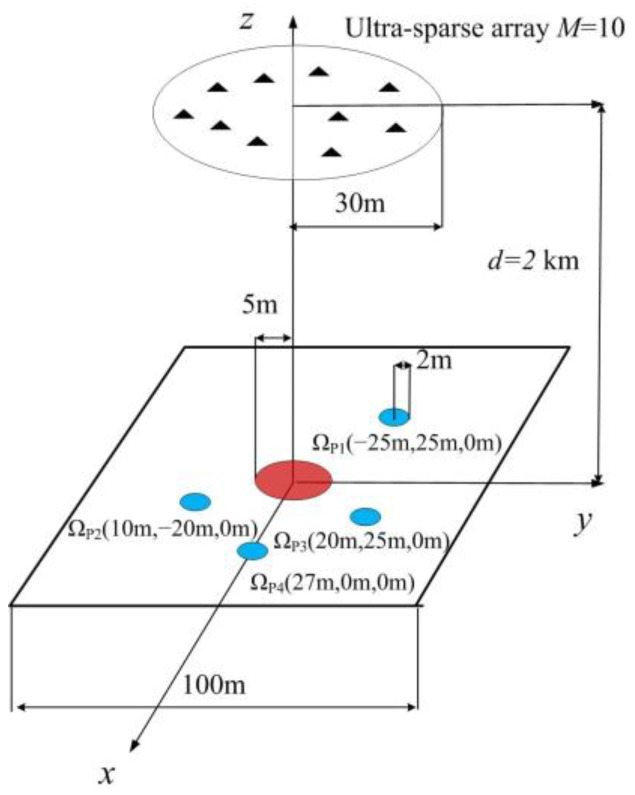
Typical DIPJ application scenario.

We verify the performance of the designed wideband jamming waveform by means of a typical DIPJ scenario, as illustrated in Figure 2. The simulation region is a 100 m × 100 m square area set in the XOY plane. The ultra-sparse array is composed of *M* = 10 transmitters randomly positioned within the circular regions with radii of 30 m at a distance *d* = 2000 m from the XOY plane. The carrier frequency was *f*_0_ = 1.0 GHz, the baseband and bandwidth were *B* = 100 MHz and *N* = 100. The grid size in the target and protected areas was 1 m × 1 m. Ω_J_ was a circular region with a radius of 5 m centered at the (x, y, z) coordinates (0 m, 0 m, 0 m), and Ω_P_ consisted of four circular regions with a radius of 2 m centered at (−25 m, 25 m, 0 m), (10 m, −25 m, 0 m), (20 m, 25 m, 0 m), and (27 m, 0 m, 0 m). The simulations were conducted using MATLAB 2019a. 

### 4.2. Comparison of Different Algorithm Effects

**Figure 3:**
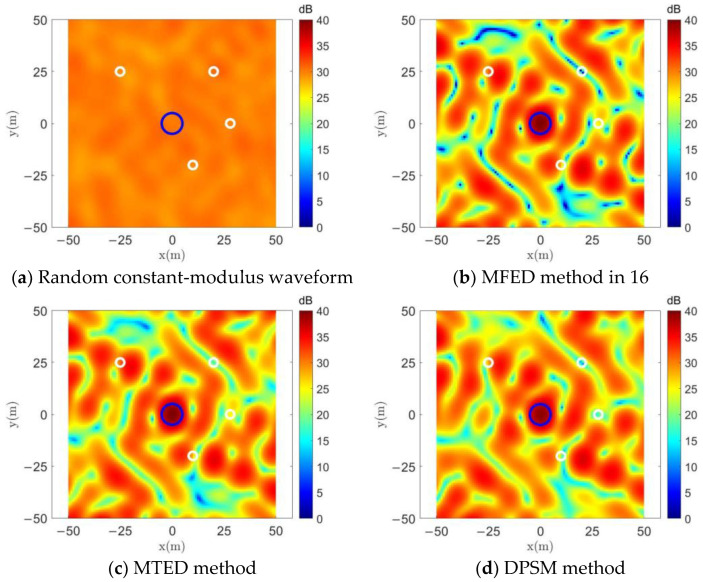
Spatial energy distribution comparison.

Firstly, the effectiveness of the jamming waveforms designed using the different algorithms is compared. Similar to in the previous studies [10,16,17,18,19,20], we normalize all the power spectrum values calculated using Equation (12) by d^2^ and convert them into a dB representation. The normalized power spectra are summed over the frequency domain by

(38) $$E_{\sigma }=\sum_{p=-N/2}^{N/2-1}P_{\sigma ,f_{p}},$$

and their spatial energy distributions are shown in Figure 3. The blue circles in the figure mark the locations of the jamming region, and the four white circles correspond to the four protected areas.

In Figure 3a, a random set of constant-modulus waveforms whose energy is presented as uniformly distributed in space is used to perform a comparison. In Figure 3b, the MFED algorithm [16] is actually solved using Equation (22). Both the MFED algorithm and the MTED method can be seen in Figure 3c to be capable of achieving energy focus in the jamming region. Since the optimization target of the DPSM method in Figure 3d is to minimize the energy in the protected region instead of maximizing the energy difference with the jamming region, it has a better energy suppression effect in the protected area than MFED or MTED, and it can also concentrate energy in the jamming area.

**Figure 4:**
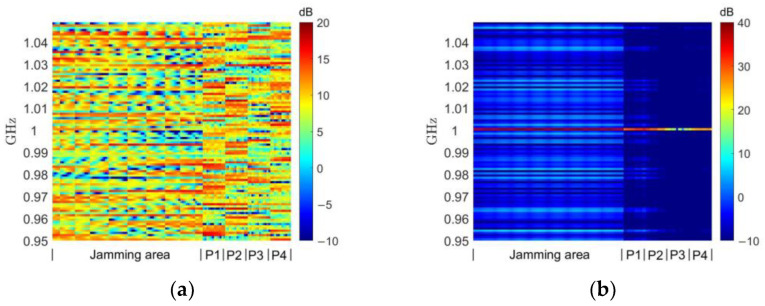

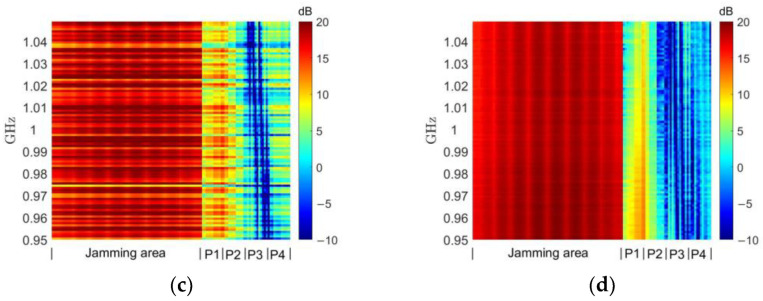
Waveform power spectrum comparison. (**a**) Random constant-modulus waveform. (**b**) MFED method in [16]. (**c**) MTED method. (**d**) DPSM method.

As discussed in Section 3, for jamming tasks, we are not only concerned with the distribution of the jamming waveform in terms of spatial energy, but also with its power spectrum. The normalized power spectra of the jamming waveforms obtained using different methods in each of the grid cells in the jamming and protected regions are given in Figure 4, where the vertical axis indicates the frequency and the horizontal axis is labeled with the grid cell of the jamming region and each protected region.

The random constant-modulus waveform shown in Figure 4a is completely random in terms of its power spectrum distribution. Because the MFED algorithm only targets total energy without considering its frequency distribution, even though its spatial energy achieves a focusing effect, as shown in Figure 3b, it can be seen from Figure 4b that the energy of its jamming waveform is mainly concentrated on only one frequency point of the power spectrum. This obviously cannot be used as a jamming waveform to cover the working bandwidth of the target to achieve a suppressed jamming effect. With our improvements, the MTED algorithm avoids concentrating the energy in a single frequency point of the power spectrum. However, as can be seen in Figure 4c, since it is optimized for a single frequency band, some amplitude information is discarded when synthesizing the constant-modulus waveform using Equation (24). This causes the power of the jamming region in some bands to not be sufficiently high, while the power in the protected region in some bands will be too high. 

With respect to the DPSM algorithm, it can be seen in Figure 4d that the jamming energy is focused throughout the frequency band, while there are no particularly high-energy points in the protected region. Note the relatively high energy in protected region 1, which is related to the grating lobe effect of the ultra-sparse array mentioned previously, and the current waveform may form a higher grating lobe precisely in protected region 1 in order to focus in the jamming region. Adjusting the randomly generated UAV position (by fixing the random number seed, several algorithms can be compared using the same UAV positions) may improve this, but our algorithm does not involve the optimization of the UAV position, which is a possible future research direction [18]. Additionally, if the weight of the scaling factor $w_{2}$ in Equation (28) is adjusted, the energy in this region could be further suppressed at the expense of partial focusing performance, as will be shown in Section 4.3.

**Figure 5:**
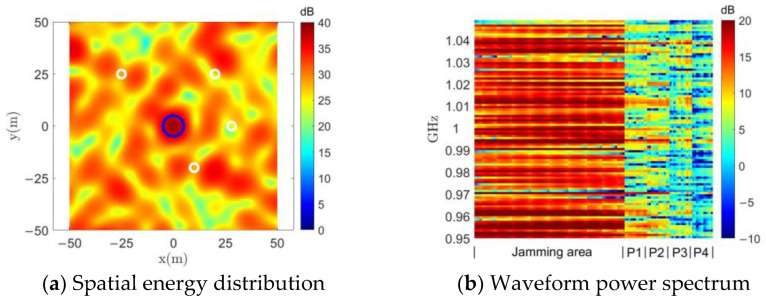
The effect of the wideband jamming waveform designed using the narrowband model method.

In particular, the effect of a set of broadband jamming waveforms is also shown in Figure 5; these were designed using the SCP (Sequential Cone Programming) algorithm [37] under the narrowband model. Here, the optimization of the SCP algorithm refers entirely to the approximate narrowband model presented in Equation (2). It can be seen from Figure 5a that the spatial energy of the jamming area is still able to produce a certain focusing effect (the quantitative results show that the spatial energy of the jamming area is about 1.5 dB lower than that obtained using the MTED algorithm, on average), but the inhibition effect on the protected area is not obvious. From the power spectrum presented in Figure 5b, it is more obviously apparent that, because the approximate model only considers the case of the central carrier frequency 1 GHz, the jamming energy periodically appears at high and low points, with 1 GHz as the center, which leads to its focus possessing low total energy. The four protected areas also have high power spectrum points in some frequency bands for the same reason.

### 4.3. Comparison of DSPM Method under Different Parameters

We verified the effectiveness of the DSPM algorithm relative to other algorithms, and in the next step we will focus on comparing the effect of jamming waveforms designed with the DSPM algorithm using different parameter settings. In Section 4.2, *w*_1_ and *w*_2_ were set to *w*_1_ = 1 − *w*_2_ and *w*_2_ = 10^−4^, respectively. In this section, we will modify *w*_2_ to 0 and 10^−3^, representing conditions under which the protected region energy is not considered to be limited, and in which the weight of the protected region is increased by a factor of 10, respectively.

**Figure 6:**
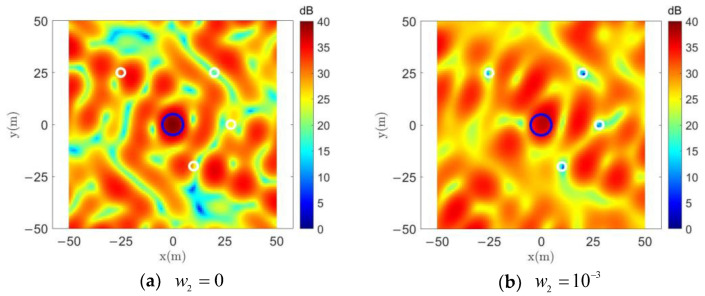
Spatial energy distribution with different *w*_2_ using the DSPM method.

**Figure 7:**
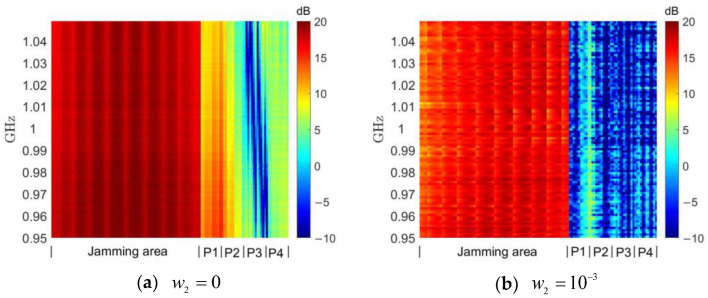
Waveform power spectrum with different *w*_2_ using the DSPM method.

It can be seen in Figure 6a and Figure 7a that with the setting *w*_2_ = 0, the energy and power spectrum of the designed jamming waveform generated in the protected region increases significantly due to the absence of suppression compared to the previous *w*_2_ = 10^−4^. As will be shown later, the average energy level in the protected region at this time is close to that of the random constant-modulus waveform. When setting *w*_2_ = 10^−3^, the energy and power spectrum of the jamming waveform in the protected region in Figure 6b and Figure 7b are very significantly suppressed compared to the previous setting of *w*_2_ = 10^−4^, but this results in a considerable sacrifice of focusing performance of the jamming region. 

In order to more clearly represent the magnitude of the power at different frequencies, we use

(39) $$JP_{f_{p}}=\sum_{\sigma \in U}P_{\sigma ,f_{p}}/I\;,\;PP_{f_{p}}=\sum_{\sigma \in V}P_{\sigma ,f_{p}}/J$$

to take the average power spectrum of all the grids in the jamming area and the protected area, respectively.

**Figure 8:**
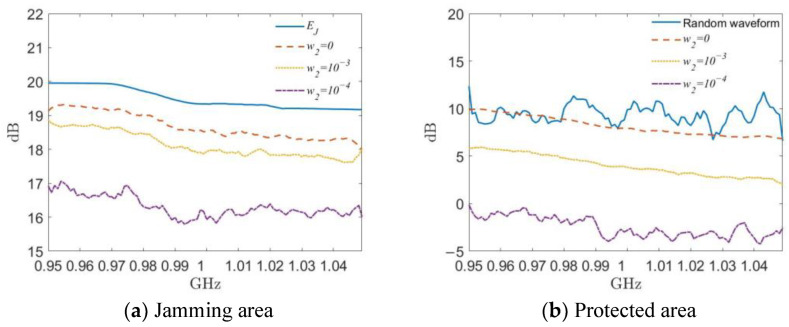
Waveform average power spectrum with different values of *w*_2_ using the DSPM method.

Setting *w*_2_ = 0 indicates the presence of no restrictions on the protected region, reflecting the best focusing effect of the DSPM method in the jamming region. As shown in Figure 8a, the average power spectrum of the jamming waveform designed under this condition lies at around 19 dB in the jamming region. This is close to but does not exceed the desired power spectrum calculated using Equation (27), which verifies the reasonableness of the desired power spectrum setting. As shown in Figure 8b, if the energy level of the protected region is not suppressed, the designed jamming waveform power spectrum will lie at around 10 dB, which is similar to the effect produced by using a random constant-modulus signal as the jamming waveform. When setting *w*_2_ = 10^−4^, the jamming waveform average power in the protected area in Figure 8b is reduced to around 5 dB, while the jamming waveform average power spectrum in the jamming area in Figure 8a only has a small reduction of less than 0.5 dB. After the value of *w*_2_ is expanded by a factor of 10 to 10^−3^, the average power spectrum of the jamming waveform in the protected region is reduced to below 0 dB in Figure 8b. The corresponding average power spectrum of the jamming waveform in the jamming region is reduced by 2 dB, and then concentrated at around 17 dB in Figure 8a. In summary, the focus of the jamming waveform design in the jamming region energy concentration and protected area suppression can be adjusted by the weight of the scaling factor, which should be decided according to the actual task requirements. 

**Figure 9:**
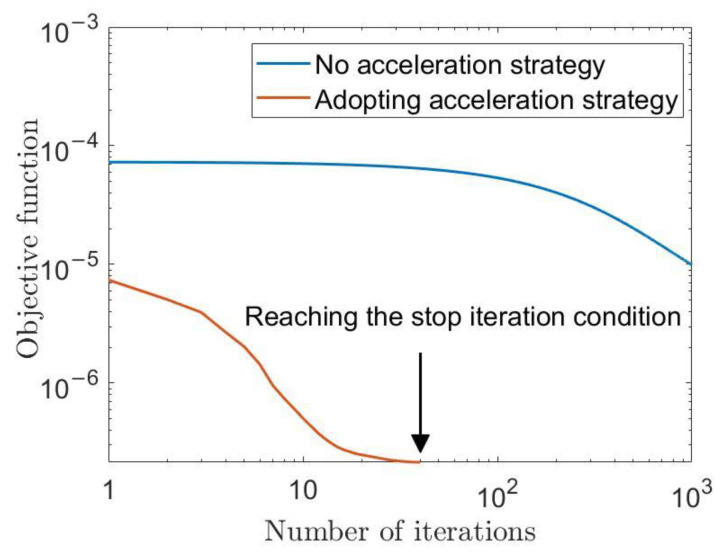
MM algorithm acceleration strategy impact.

Figure 9 shows the effect of the acceleration strategy of the MM algorithm in Section 3.2.3. Since the MM algorithm is deterministically convergent, we set the threshold for stopping the iterations ε to be equal to the difference between the objective functions of two iterations less divided by the initial objective function less than 10^−5^. The convergence condition is reached after about fifty iterations of the DSPM method with the acceleration strategy (note that, as shown in Algorithm 3, the MM algorithm is executed twice for each iteration with the acceleration strategy), while the corresponding DSPM method without the acceleration strategy is executed 1000 times and is still far from reaching the stopping condition. As discussed in Section 3.2, the computational complexity of the DSPM method is related to the number of iterations, and the acceleration strategy greatly reduces the computational complexity of the MEDP algorithm. 

**Figure 10:**
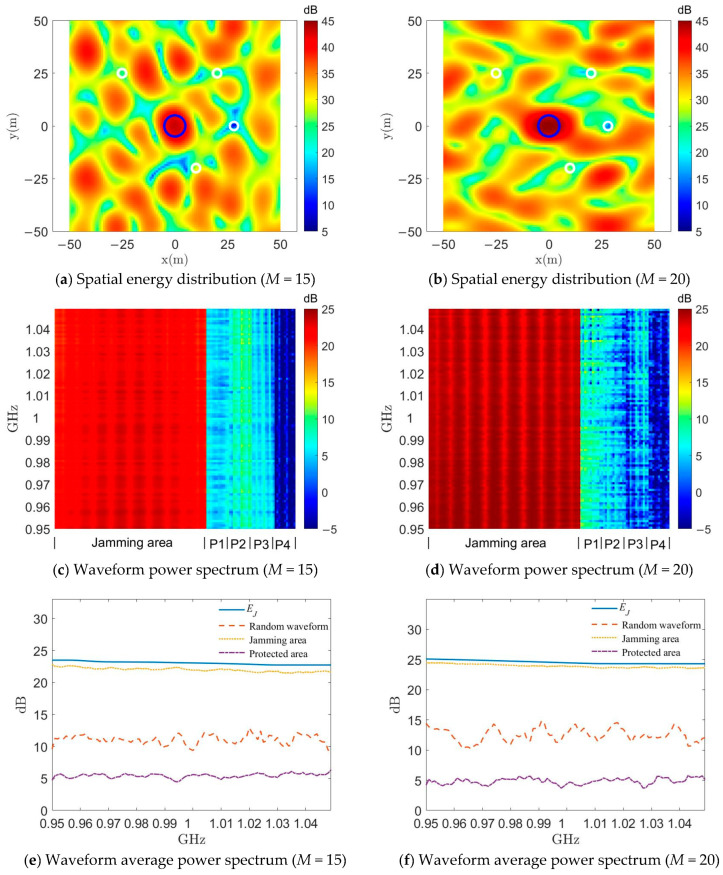
Comparison of the effect of the DSPM method with different numbers of UAVs.

Figure 10 shows the change in the values of the designed jamming waveform after fixing *w*_2_ = 10^−4^ but increasing the number of UAVs to 15 and 20 (note: the range of energy values shown in the graph is different from that in the previous figures due to the increase in overall launch energy). One point of interest is that the total energy emitted at *M* = 20 is only 3 dB higher than that at *M* = 10, as evidenced by the increase in the average power spectrum of the random constant-mode waveform shown in Figure 10e from about 9.5 dB to about 12.5 dB. However, the average power spectrum of the jamming waveform in the jamming region is increased by about 5 dB, which indicates directly that the effect of energy focusing increases significantly when the number of UAVs is increased. In the future, if large-scale UAV clusters are to be used for jamming missions, the significance in the implementation of DIPJ studied in this paper will be more prominent when the jamming suppression effect needs to be enhanced as much as possible because of the limited emission energy of individual UAVs. Another interesting phenomenon is that, despite increasing the number of UAVs is increased, the designed jamming waveform nevertheless does not significantly increase the energy or average power spectrum in the protected area. This may be due to the increase in the number of UAVs in the fixed area attenuating the sparsity of the transmitter arrangement, and thus reducing the grating lobe effect, which is worthy of further study in the future. 

Finally, we give the calculation time of a single iteration of the MTED and DSPM algorithms under the condition of the number of UAVs *M* and the length of waveform *N* under different parameters in Table 1. Please note that one iteration time of the MTED algorithm here refers to the total operation time of all *N* frequency points. The computational complexity of the two algorithms is related to *N*^3^ and *M*^2^, so when *N* increases, the impact on the calculation time is significantly greater than when *M* increases, which is consistent with our computational complexity analysis. Although the optimization effect of the previous MTED algorithm is weaker than that of the DSPM algorithm, its lower computation time is its advantage, which is also in line with the computational complexity analysis results of our previous algorithm.

## 5. Conclusions

In this paper, we tried to solve the DIPJ jamming waveform design problem by means of a wideband signal model. First, the previous mathematical model based on narrowband signals was extended in order to consider wideband situations. Following the simple improvement of the previous algorithm, the MTED method was determined, which is applicable to the broadband model and has low computational complexity. In order to obtain a jamming waveform with better effect, we proposed the DSPM method and provided a detailed derivation. To address the lack of matching targets when using the DSPM method, a design method was developed for the desired power spectrum, while an approximate solution method for the maximum eigenvalue and an iterative acceleration strategy were developed in order to reduce the computational complexity of the DSPM method in the iterative process of the MM algorithm. A typical DIPJ scenario was used to verify the effectiveness of the algorithm. The quantitative results showed that the jamming waveform designed using the DPSM and MTED method was able to solve wideband DIPJ tasks that could not be handled by the jamming waveforms designed using the previous narrowband algorithm. Compared with the MTED method, DPSM has better performance and the parameters can be flexibly adjusted. The disadvantage of DPSM is that the computational complexity is greater, even when using the acceleration algorithm. Our next research direction will be to design a lower-complexity method on the premise of ensuring the performance of jamming waveform. At the same time, the experiment shows that an improved DIPJ effect can be obtained by increasing the number of UAVs, but the problem of collaborative management prompted by the increase in the number of UAVs needs to be studied and is also a problem worth discussing at the next research stage. 

## Figures and Tables

**Table 1:** Comparison of computation time for a single iteration between proposed algorithms.

*M*	*N*	MTED	DPSM
10	50	0.3 s	0.8 s
	100	1.7 s	4.3 s
	150	4.7 s	9.4 s
	200	10.1 s	23.2 s
5	100	0.7 s	1.1 s
15		3.1 s	6.4 s
20		4.9 s	10.8 s

## Data Availability

Data sharing not applicable due to institutional limitations.
